# Hydroxychloroquine modulates the progression of experimentally induced benign prostatic hyperplasia in rats via targeting EGFR/ERK/STAT3 and AR/FOXO1/TRAIL pathways: computational and in vivo studies

**DOI:** 10.1038/s41598-025-04267-y

**Published:** 2025-06-20

**Authors:** Walaa H. El-Maadawy, Ehab Hafiz, Samer A. Tadros, Sally A. Fahim, Haidy M. Ebrahim, Marwa A. Fouad, Yasmin M. Attia

**Affiliations:** 1 Pharmacology Department, Theodor Bilharz Research Institute, Warrak El-Hadar, Imbaba, P.O. box 30, Giza, 12411 Egypt; 2 Electron Microscopy Department, Theodor Bilharz Research Institute, Warrak El-Hadar, Imbaba, P.O. box 30, Giza, 12411 Egypt; 3 Department of Biochemistry, Faculty of Pharmacy, October University for Modern Sciences and Arts (MSA), 26 July Mehwar Road Intersection with Wahat Road, 6th of October City, P.O. Box 12451, Cairo, Egypt; 4 Department of Biochemistry, School of Pharmacy, Newgiza University (NGU), Newgiza, km 22 Cairo-Alexandria Desert Road, P.O. Box 12577, Giza, Egypt; 5 Animal House Unit, National Cancer Institute, Cairo University, Kasr Al Eini Street, Fom El Khalig, Cairo, Egypt; 6 Department of Pharmaceutical Chemistry, Faculty of Pharmacy, Newgiza University (NGU), Newgiza, km 22 Cairo-Alexandria Desert Road, Cairo, Egypt; 7 Pharmaceutical Chemistry Department, Faculty of Pharmacy, Cairo University, Kasr El-Aini St., P.O. Box 11562, Cairo, Egypt; 8 Pharmacology Unit, Cancer Biology Department, National Cancer Institute, Cairo University, Kasr Al Eini Street, Fom El Khalig, Cairo, Egypt

**Keywords:** Benign prostate hyperplasia, Hydroxychloroquine, EGFR, STAT3, FOXO1, TRAIL-mediated apoptosis, Histocytochemistry, RNA, Biochemistry, Gastroenterology, Pathogenesis, Urology

## Abstract

Benign prostatic hyperplasia (BPH) is a prevalent progressive age-related disorder in men, yet its etiopathophysiology remains poorly understood. Current treatments like finasteride (Fin) have limited long-term efficacy, necessitating alternative therapies. Hydroxychloroquine (HCQ), a safe antimalarial agent, possesses anti-inflammatory, immunomodulatory, and antiproliferative activities, however, its therapeutic effect in BPH has not been investigated. Accordingly, we examined its therapeutic potential and underlying mechanisms, alone or combined with Fin, in testosterone-induced BPH in rats. In BPH-induced rats, HCQ markedly reduced prostate weight and index, and PSA, testosterone, dihydrotestosterone, pro-inflammatory cytokines (TNF-α, κ and IL-6), and the transcription factor “NF-κB” levels, while improving histological abnormalities in epithelial and stromal tissues. HCQ reduced the mRNA expression of AR and ERK1/2, and decreased the protein levels of EGFR and STAT3. Additionally, HCQ increased the mRNA expression of FOXO1 and promoted apoptosis through both intrinsic and TRAIL-mediated pathways. This was evidenced by the upregulation of pro-apoptotic Bax and the downregulation of anti-apoptotic Bcl-2 and Bcl-XL levels in the intrinsic pathway, as well as the reduction in mRNA expression of DR4 and DR5 in the TRAIL-mediated pathway. Notably, combining HCQ with Fin enhanced these effects. Molecular docking revealed HCQ’s strong interactions with androgen receptor (AR), EGFR, ERK1/2, FOXO, and TRAIL death receptors (DR4/DR5), comparable to Fin except for STAT3. Our findings suggest that HCQ modulates BPH progression by targeting STAT3/FOXO1/TRAIL and EGFR/ERK/AR pathways, offering a promising therapeutic strategy for BPH, either alone or in combination with Fin.

**Supplementary Information:**

The online version contains supplementary material available at 10.1038/s41598-025-04267-y.

## Introduction

Benign prostatic hyperplasia (BPH) is a benign progressive enlargement of the prostate gland. It is defined by the excessive and uncontrolled proliferation of epithelial and fibromuscular tissues in the transition zone and periurethral region^1^. It is considered an immune inflammatory disease characterized by prostatic hyperplasia and lower urinary tract symptoms (LUTS)^2^. The occurrence of BPH rises after reaching the age of 40, with a prevalence ranging from 8 to 60% by the time individuals reach 90 years old^3^. In 2019, the global number of individuals aged 60 and older affected by BPH reached 79 million^4^.

Despite its high prevalence and socioeconomic impact, the etiopathophysiology of BPH is not completely elaborated. The lack of a definitive foundation for hyperplasia hinders efforts to create novel treatments^5^. Notably, the dihydrotestosterone (DHT)-mediated pathway is considered the most common and well-documented mechanism in BPH pathogenesis^6^. DHT, the tissue-active form of testosterone, acts on the androgen receptor (AR) inducing prostate stromal^7^ and epithelial cell growth with enhanced epithelial-mesenchymal transition (EMT)^8^. Given this mechanism, the first-line treatments, such as dutasteride and finasteride (FIN), act by inhibiting the 5α-reductase enzyme, preventing the conversion of testosterone to DHT^9^. In addition, several signaling pathways have similarly been proposed to participate in BPH development especially those related to apoptosis, and inflammation. These reported pathways include the epidermal growth factor receptor (EGFR) signaling, which initiates downstream cascades of chronic inflammation, proliferation, and apoptosis inhibition in prostatic tissues^10–12^. Additionally, Signal Transducer and Activator of Transcription (STAT)3 and nuclear factor (NF)-κB, are major transcription factors in prostatic inflammation, playing essential roles in BPH progression^12,13^. The AR/ Forkhead Box O (FOXO)1 axis, another key regulator of prostatic growth, improves BPH progression when regulated^14–16^.

Nevertheless, pharmacological treatments may lose efficacy over time, often necessitating surgical intervention in selected cases^17^. Also, BPH drugs like Fin are linked to several side effects, including reduced libido, erectile dysfunction^9^, and in some cases depression, gynecomastia^18,19^, and orthostatic hypotension^20^. Over the past two decades, several minimally invasive procedures such as transurethral microwave thermotherapy, UroLift, and prostate artery embolization have emerged as alternatives to conventional surgery. However, many of these techniques have been largely abandoned, attributed to their ineffectiveness and need for retreatment^5^. Combination therapies, on the other hand, have demonstrated higher efficacy, fewer side effects, and improved BPH patients’ quality of life. Tamsulosin, an α-1 A adrenergic receptor blocker, enhances therapeutic outcomes and delays BPH progression when combined with FIN^21^.

Hydroxychloroquine (HCQ) has been a safe antimalarial agent for many years. It is currently employed as monotherapy or combined with other therapies for treating autoimmune conditions, such as systemic lupus erythematosus and rheumatoid arthritis, due to its potent anti-inflammatory effects^22^. Several clinical studies suggested that HCQ could also be combined with other chemotherapeutic agents for treating various cancers, including prostate cancer^23–25^. This versatility stems from its unique pharmacokinetic profile and multiple mechanisms of action^26^. HCQ modulates several signaling pathways, including EGFR^27^, STAT3^28^, and FOXO signaling^29^ as well as exhibiting antiproliferative and apoptotic-inducing activities^30,31^ in cancer therapy and various illnesses. Such multifaceted mechanisms make HCQ a promising candidate in the treatment of BPH.

Consequently, the objective of this study was to explore the therapeutic effectiveness of HCQ, both alone and in combination with FIN, in alleviating testosterone-induced BPH in rats, while also revealing the molecular mechanisms at play. Moreover, computational studies, including molecular docking and protein-protein interactions, were performed to validate HCQ or FIN as potential modulators of AR/FOXO1/TRAIL and EGFR/ERK/STAT3 signaling pathways.

## Materials and methods

### Drugs & reagents

Testosterone enanthate (TE) (Cidoteston^®^), HCQ (Plaquenil^®^), and Fin (Prostride^®^) were purchased from Chemical Industries Development Co. (CID) Pharmaceuticals, Sanofi Aventis Pharmaceuticals, and ADWIA Pharmaceuticals, Egypt, respectively. Isoflurane (AErrane^®^) was purchased from Baxter (Bielefeld, Germany). Carboxymethyl cellulose (CMC) and hematoxylin and eosin (H&E) stains were procured from Sigma-Aldrich, MO, USA. Phosphate buffer saline (PBS) and BCA protein assay kit were procured from Lonza Bioproducts, Verviers, Belgium, and Bio Basic Inc., Markham Ontario, Canada, respectively. Enzyme-linked immunosorbent assay (ELISA) kits for testosterone, DHT, Interleukin (IL)-6, tumor necrosis factor (TNF)-α, and NF-κB were purchased from Abbexa Ltd., Cambridge, UK, while Prostate-Specific Antigen (PSA), Bax, Bcl2 and Bcl2-XL were procured from Cusabio Biotech Co. Ltd., Houston, TX, USA. Easy-spin RNA extraction kit and SYBR Green PCR Master Mix were obtained from Intron Biotechnology, Korea. cDNA and primers were procured from Thermo Fisher Scientific, NY, USA. The primary antibodies against STAT3 (#9139T), pSTAT3 (#73533SF), EGFR (#4267T), pEGFR (#3777T), and horseradish peroxidase (HRP)-conjugated secondary antibodies (#58802S and #7074P2) were obtained from Cell Signaling Technology, CA, USA. The other chemicals used were of the highest commercially available quality.

### Animals and experimental design

Thirty adult male Sprague-Dawley rats (weighing 300–350 g), were purchased from the animal house of the National Cancer Institute. Rats were kept on a 12 h light/dark cycle, controlled humidity and temperature at 23 ± 2 °C, and provided free access to food and water *ad libitum*. The experiment was initiated following the approval of the research protocol by the Research Ethics Committee of Theodor Bilharz Research Institute (PT 852, 25/7/2024) as per the National Institutes of Health (eighth edition) guidelines for laboratory animal care and use, and in adherence with the ARRIVE guidelines. All experimental procedures were performed by appropriately trained, qualified, and competent personnel with FELASA training.

**Fig. 1:**
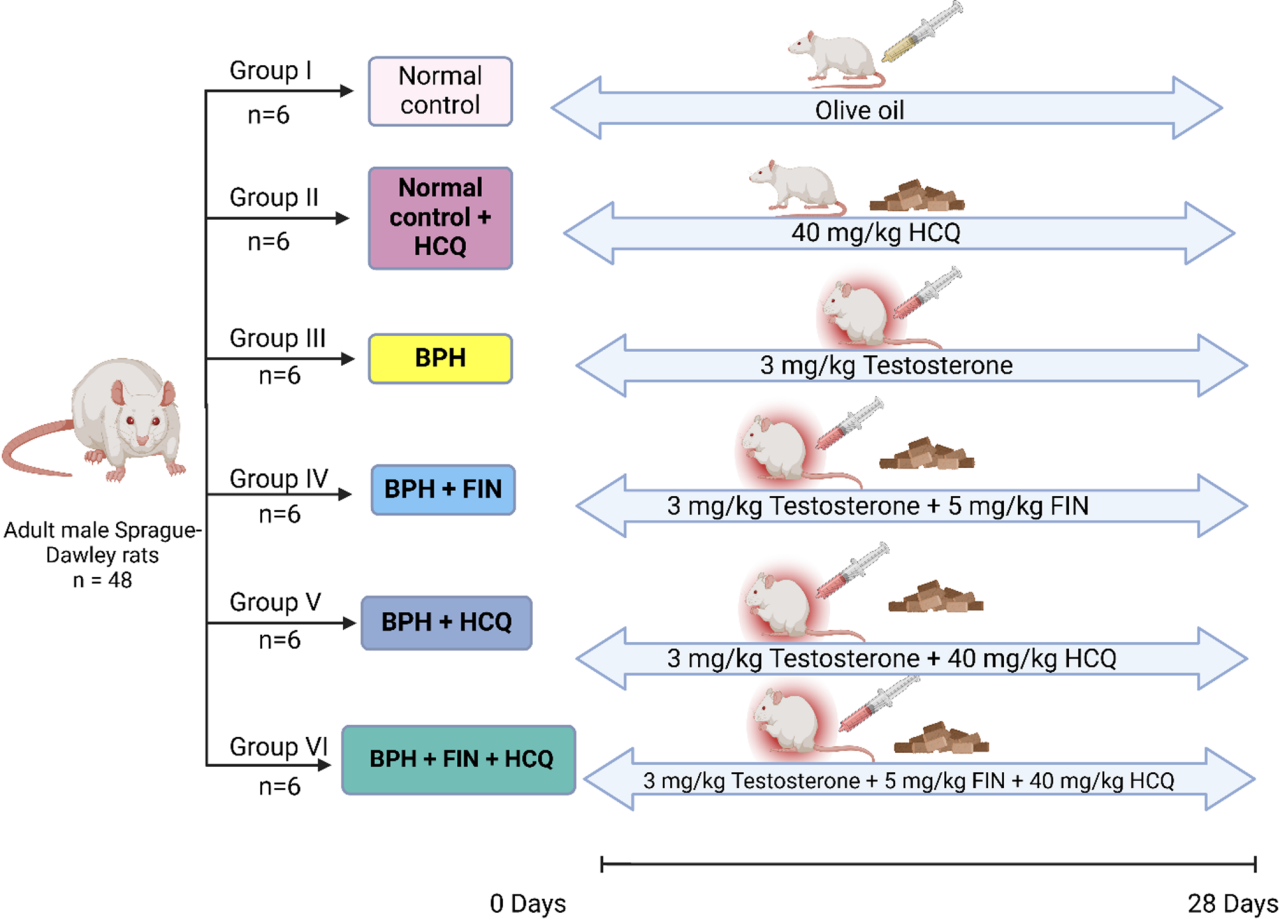
The experimental design and animal experiment flow chart.

Rats were randomly allocated into six groups (*n* = 6 rats/group) following a week of acclimatization, as follows: **Group I (Normal control)**: normal rats were given 0.5% CMC, concurrently with SC injection of olive oil as drug vehicles, **Group II (Normal control + HCQ)**: normal rats were administered 40 mg/kg HCQ^32^, **Group III (BPH)**: rats were SC injected TE (3 mg/kg) diluted in olive oil to induce BPH^33^, **Group IV (BPH + Fin)**: rats were administered 5 mg/kg Fin^34^ concurrently with testosterone, **Group V (BPH + HCQ)**: rats were orally administered 40 mg/kg HCQ concurrently with testosterone, and **Group VI (BPH + HCQ + Fin)**: rats will be orally administered a combination of HCQ (40 mg/kg) and Fin (5 mg/kg) concurrently with testosterone. All drugs and vehicles were orally administered once per day for 28 consecutive days, and a schematic diagram of treatments with dosage schedules is shown in Fig. 1. At the end of the experiment, rats were weighed, and blood samples were collected from the retro-orbital plexus and centrifuged at 4000 rpm for 15 min. Sera were then separated and stored at − 80 °C for subsequent analyses. Next, rats were sacrificed by cervical dislocation under light isoflurane anesthesia. The prostates were dissected and weighed, and the ventral lobes were fixed in 10% formalin for histopathological examinations. The remaining prostatic tissues were snap-frozen and stored at –80 °C for further biochemical, RT-PCR, and western blot investigations.

### Prostate weight (PW) and prostate index (PI)

The PI is calculated as the relative PW to body weight ratio and the percent inhibition of PI were determined using the following equations^35^:

$$(I)PI=PW \div Body\;weight$$

$$\begin{aligned} (II)\;Percent{\text{ }}inhibition & ={\text{ 1}}00 - \{ (treated\;group - normal) \\ & \;\;\;\; \div (BPH\;group - normal){\text{ }} \times {\text{ 1}}00\} \\ \end{aligned}$$

### ELISA

The serum levels of PSA, testosterone, and DHT were quantified according to the manufacturer’s instructions. Moreover, the levels of pro-inflammatory (IL-6, TNF-α, and NF-κB) and apoptotic (Bax, Bcl2, and Bcl2-XL) markers were determined in prostatic tissues as per the manufacturer’s instructions. The protein content/prostate tissue was quantified according to the Bradford method.

### Western blot analysis

The prostatic tissues were rinsed with saline, dried, and then homogenized. The homogenates underwent centrifugation at 10,500 g for 20 min at 4 °C. Subsequently, protein concentrations were determined in the medium and cell lysate using the Bradford method as previously described^36^. Anti-STAT-3, anti-p-STAT3, anti-EGFR, anti-p-EGFR were used. An HRP-linked secondary antibody was utilized at a dilution of 1:5000. The chemiluminescent substrate (ClarityTM Western ECL substrate, Bio-Rad cat#170–5060) was applied to the blot. Image analysis software was employed to assess the band intensity relative to the control sample β-actin (a housekeeping protein) through protein normalization on the ChemiDoc MP image.

### Quantitative real-time polymerase chain reaction (qRT-PCR)

The total RNA was isolated from tissue homogenate with an RNA extraction kit, and cDNA was obtained. The cDNA was then amplified by PCR with primers for AR, death receptor (DR)4, DR5, ERK1/2, and FOXO1, and a reference housekeeping gene (β-actin). The prepared reaction mix samples were applied in real-time PCR (StepOne Applied Biosystem, Foster City, USA) using SYBR Green PCR Master Mix. The primer sequences are shown in Table 1. Quantitative data analysis was done as previously described by Livak & Schmittgen^37^. Values are displayed as relative expression levels.

**Table 1:** Primer sequences for RT-qPCR.

Gene symbol	Primer sequence from 5′- 3′
AR	F: CTGATTCCTTTGCTGCCTTGT R: ATTAGTGAAGGACCGCCAACC NM_012502.2
DR4	F: TGATGAAGAGTGCCAGAAATAGC R: CCAGGTCCATCAAATGCTCA NM_145681.2
DR5	F: AAATGCTGCTGAAGTGGCT R: ACTAATAAAGATCCTCTCGGCTC NM_001108873.1
ERK1/2	F: ACGGCATGGTCAGCTCAGC R: ATCCGAGACATCCTCAGAG XM_039088525.2
FOXO1	F: GAT AAG GGC GAC AGC AAC AG R: TGA GCA TCC ACC AAG AAC TT NM_001191846.3
β-actin	F: TCCGTCGCCGGTCCACACCC R: TCACCAACTGGGACGATATG Gene bank accession number: NM_031144.3

### Histopathological examinations

The excised prostates were grossly examined for size, areas of hemorrhage, and necrosis. Samples from the ventral lobes were promptly fixed in a 10% formalin solution, processed, and embedded in paraffin. Thin sections of 4 microns were then stained with H&E stain for histological assessment of glandular and stromal alterations.

### Molecular Docking studies

The X-ray crystal structures of EGFR (PDB ID: 1M17)^38^ in complex with erlotinib, ERK1 (PDB ID: 4QTB)^39^ in complex with the inhibitor SCH772984, ERK2 (PDB ID: 6SLG)^40^ in complex with Tizaterkib (AZD0364), AR (PDB ID: 1T7R)^41^ in complex with 5-α-dihydrotestosterone, STAT3 (PDB ID: 6NJS)^42^ in complex with SD-36, FOXO1 (PDB ID: 3CO7)^43^, DR4-TRAIL (PDB ID: 5CIR)^44^, and DR5-TRAIL (PDB ID: 1D4V)^45^, were downloaded from the Protein Data Bank (https://www.rcsb.org/).

All the molecular docking studies were performed using Molecular Operating Environment (MOE, 2022.02) software. Before protein preparation, only one chain from the downloaded protein was kept. Unnecessary ligands and ions were removed, keeping the co-crystallized inhibitor. Water molecules were kept in cases where their involvement in interactions is essential, as reported for EGFR, ERK1/2, AR, and STAT3, otherwise, it was removed^38–42^. Then, the protein preparation was conducted using the default settings in MOE, employing the QuickPrep protocol. The software utilized for energy minimization was the same until reaching a root-mean-square deviation (RMSD) gradient of 0.1 kcal mol − 1 Å−1, employing the Amber 10: EHT force field. The partial charges were automatically calculated. The placement method used was Triangle Matcher, with the scoring function being London dG and the refinement scoring function being GBVI/WSA dG. To validate the docking protocol, the co-crystallized ligands were re-docked into the binding site using the abovementioned settings. The SMILES of R- and S-enantiomers of HCQ in addition to FIN were copied from PubChem (https://pubchem.ncbi.nlm.nih.gov/), sketched in Molecular Operating Environment (MOE, 2022.02) software and prepared for docking by energy minimization and partial charges optimization. The validated protocols were employed to dock the three prepared structures, and subsequently, the binding interactions of these compounds within the binding pocket were examined to anticipate their potential binding mode.

### Protein-protein interactions

The STRING protein-protein interaction database^46^ was used to examine the interconnectivity within the chosen targets of HCQ.

### Statistical analyses

Statistical analyses were conducted using GraphPad Software V 9.0 (San Diego, CA, USA). Normality was assessed using the Kolmogorov–Smirnov test. One-way analysis of variance (ANOVA) test followed by Tukey’s *post* *hoc *test was applied for statistical analyses. Statistical differences were considered significant when P < 0.05. The combination index (CI) was computed to determine the characteristics of FIN and HCQ combinations as previously outlined^47,48^. CI values below 1, above 1, and equal to 1 indicate a synergistic, antagonistic, and additive effect, respectively.

## Results

### Effect of HCQ, FIN or their combination on body weight, prostate weight, and relative prostate weight

The PW and PI were markedly increased by 3.2- and 2.2-fold, respectively, following testosterone injection in the BPH group relative to the normal control group (*P <* 0.0001). FIN, HCQ, or their combination treatment inhibited this upsurge in the PW and PI and showed a significant decrease compared to the BPH group at *P* < 0.05, < 0.01, and < 0.001, respectively. Moreover, combination-treated rats were more effective by further reducing the PW and PI compared to both FIN and HCQ groups (*P* < 0.001). However, FIN and HCQ did not differ significantly in terms of PW and PI (Table 2).

**Table 2:** Effect of HCQ and FIN or their combined administration on the PW and PI in testosterone-induced BPH in rats.

	BW (g)	PW (g)	% Inhibition of PW	PI *10^3^	% Inhibition of PI
Normal Control	310 ± 31.11	1.13 ± 0.26	-	3.72 ± 1.21	-
Normal Control + HCQ	323.33 ± 59.34	0.85 ± 0.42	-	2.62 ± 1.04	-
BPH	437.6 ± 25.96	3.69 ± 0.14^a***b***^	-	8.4 ± 0.21^a***b***^	-
BPH + FIN	432.25 ± 38.82	2.63 ± 0.24^a***b***c**^	172.85	6.09 ± 1.08^a*b***c*^	71.52
BPH + HCQ	396.6 ± 18.92	2.36 ± 0.15^a*b***c**^	180.11	5.96 ± 0.18^a*b**c*^	73.05
BPH + FIN + HCQ	370 ± 45.82	0.56 ± 0.38^c***d***e***^	228.99	1.52 ± 1.08^c***d***e***^	125.77

The values are expressed as Mean ± S.D. *: Statistically significant at *P* < 0.05, **: Statistically significant at *P* < 0.01, ***: Statistically significant at *P* < 0.001.

a: significantly different from Normal Control.

b: significantly different from Normal Control + HCQ group.

c: significantly different from BPH group.

d: significantly different from BPH + FIN group.

e: significantly different from BPH + HCQ group.

### Effect of HCQ, FIN or their combination on serum testosterone, DHT, and PSA levels, and AR gene expression

**Fig. 2:**
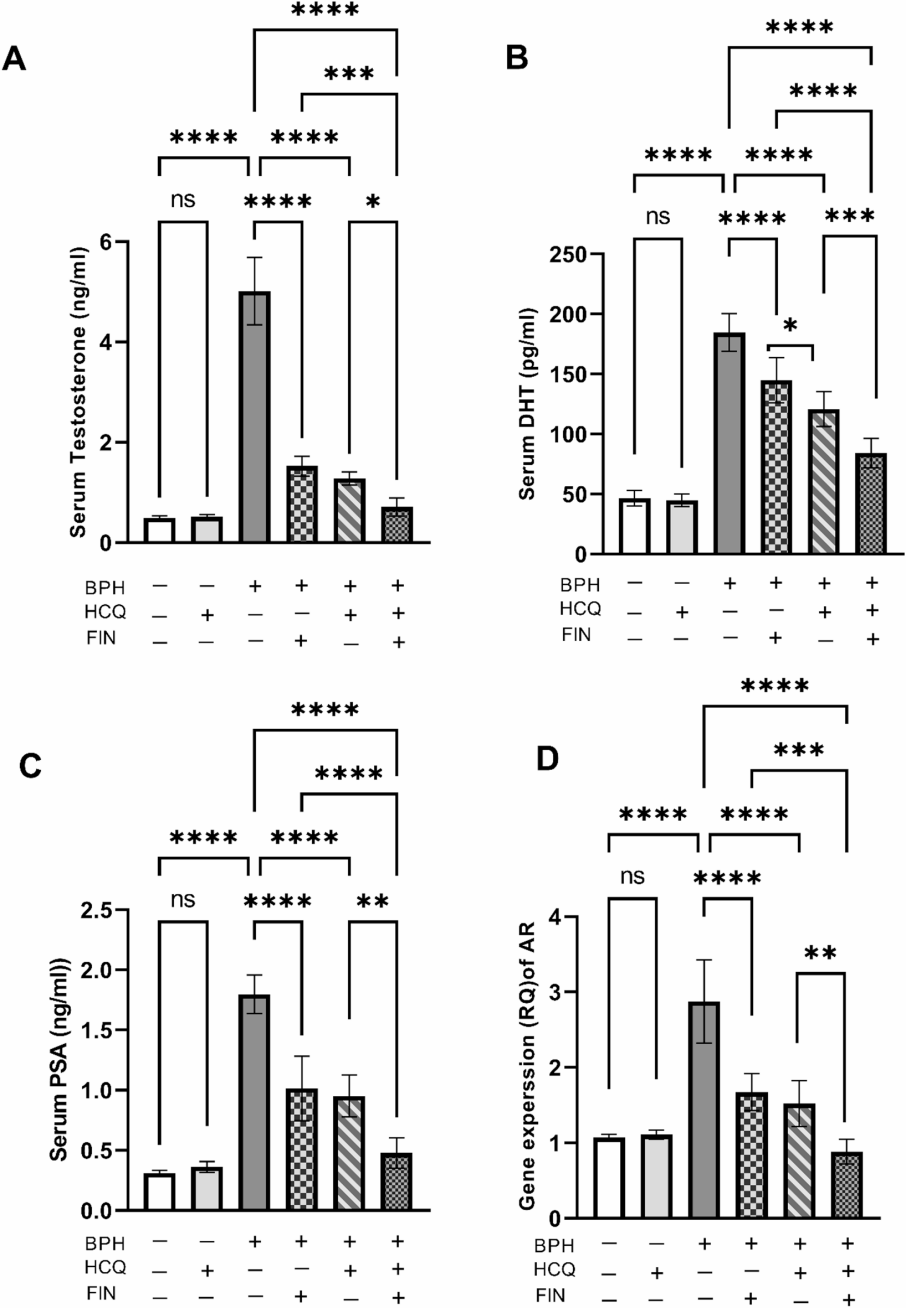
Effect of FIN, HCQ, and a combination of both on the serum testosterone (A), DHT (B), PSA (C), and AR (D) levels. Each bar with a vertical line indicates mean ± SD. One-way ANOVA followed by Tukey’s multiple comparison test was used for statistical analysis. *: Statistically significant at *P* < 0.05, **: Statistically significant at *P* < 0.01, ***: Statistically significant at *P* < 0.001.

Serum Testosterone and DHT are the main triggers of BPH, while PSA functions as a biomarker indicating BPH progression. Levels of testosterone, DHT, and PSA, and mRNA expression of AR were significantly elevated by 10-, 3.97-, and 5.23- and 2.6-fold, respectively, in the BPH group relative to the normal control group with *P* < 0.001. Treating rats with FIN, HCQ or their combination significantly reduced testosterone, DHT, and PSA levels, and AR mRNA expression. Notably, combination therapy restored PSA levels to baseline, comparable to the control group (Fig. 2). Furthermore, the combination therapy exhibited a synergistic effect with a CI < 1; registering at 0.8 for DHT levels, 0.87 for PSA levels and 0.88 for AR mRNA expression, indicating that the FIN/HCQ combination offers a more effective treatment regimen than individual therapies in impeding BPH progression.

### Effect of HCQ,  FIN or their combination on FOXO1 expression, apoptotic markers, and death receptor signaling in BPH

**Fig. 3:**
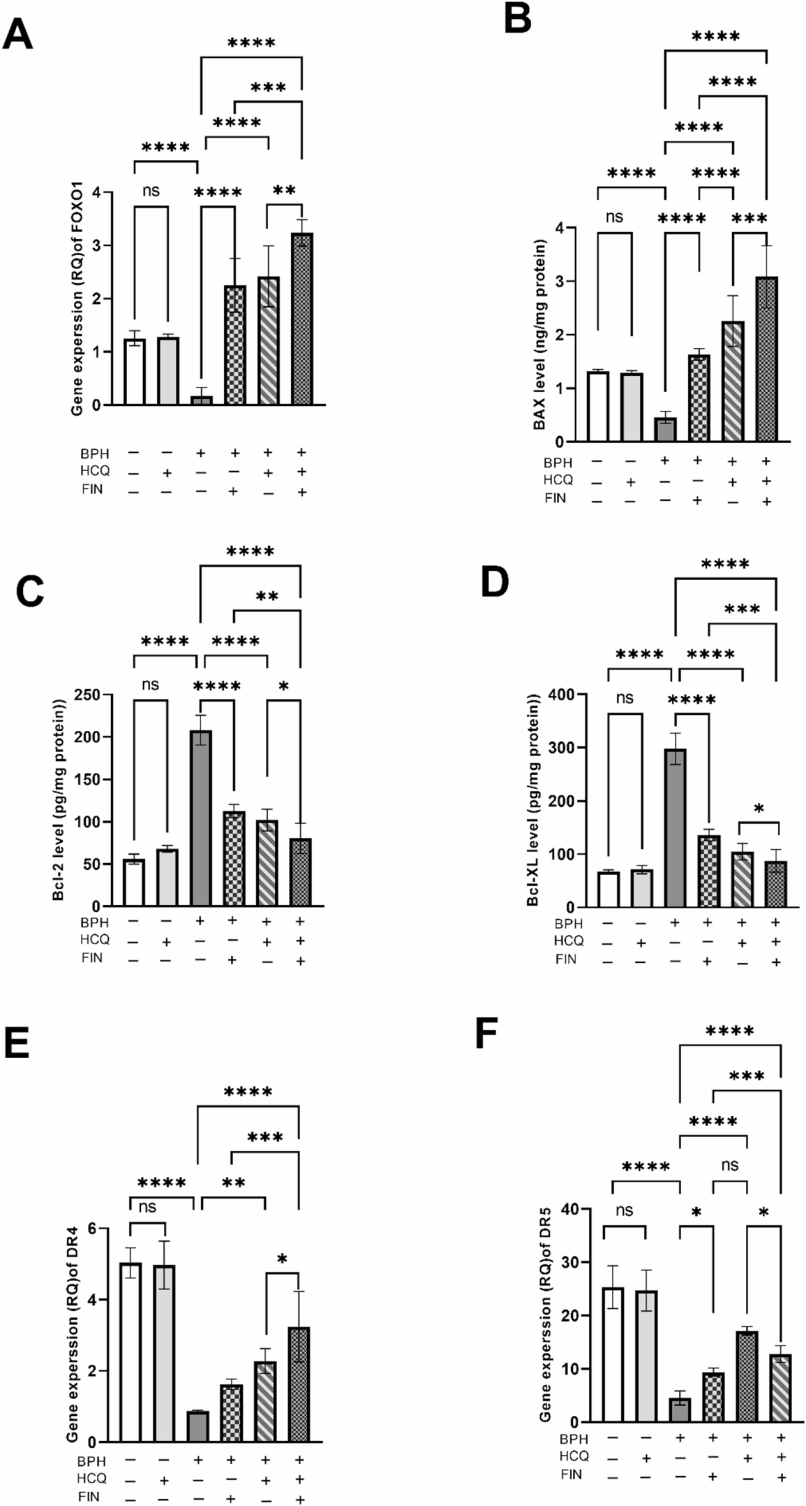
Effect of FIN, HCQ, and a combination of both on the gene expression level of FOXO (A), Bax (B), Bcl2 (C), Bcl-XL (D), DR4 (E), and DR5 (F) in prostatic tissue. Each bar with a vertical line indicates mean ± SD. One-way ANOVA followed by Tukey’s multiple comparison test was used for statistical analysis. *: Statistically significant at *P* < 0.05, **: Statistically significant at *P* < 0.01, ***: Statistically significant at *P < 0.001.*

The apoptotic signaling pathways FOXO1/Bcl2/Bax/TRAIL were measured due to their role in modulating abnormal growth in BPH. FOXO1 expression is significantly reduced in BPH groups compared to controls, however the treatment with HCQ, FIN or their combination increases its expression, with the combination showing the highest effect (Fig. 3A). Moreover, BAX, a pro-apoptotic marker, is downregulated in BPH and upregulated with HCQ or FIN, with the combination having the highest impact (Fig. 3B). On the other hand, the anti-apoptotic markers Bcl-2 and Bcl-XL are elevated in BPH but significantly reduced by HCQ or FIN, with the combination showing a stronger effect (Fig. 3C and D). Death receptor genes DR4 and DR5 are downregulated in BPH and restored with HCQ or FIN treatment, with the combination producing the most significant increase (Fig. 3E and F). Moreover, FIN/HCQ combination shows a synergistic effect with CI of 0.1, 0.38, 0.76 and 0.64 for FOXO, BAX, DR4 and DR5 respectively, underscoring the significance of this combined therapeutic approach. These findings suggest that the combination of HCQ and FIN effectively modulates key signaling pathways implicated in BPH pathophysiology.

### Effect of HCQ, FIN or their combination on inflammatory markers in BPH

**Fig. 4:**
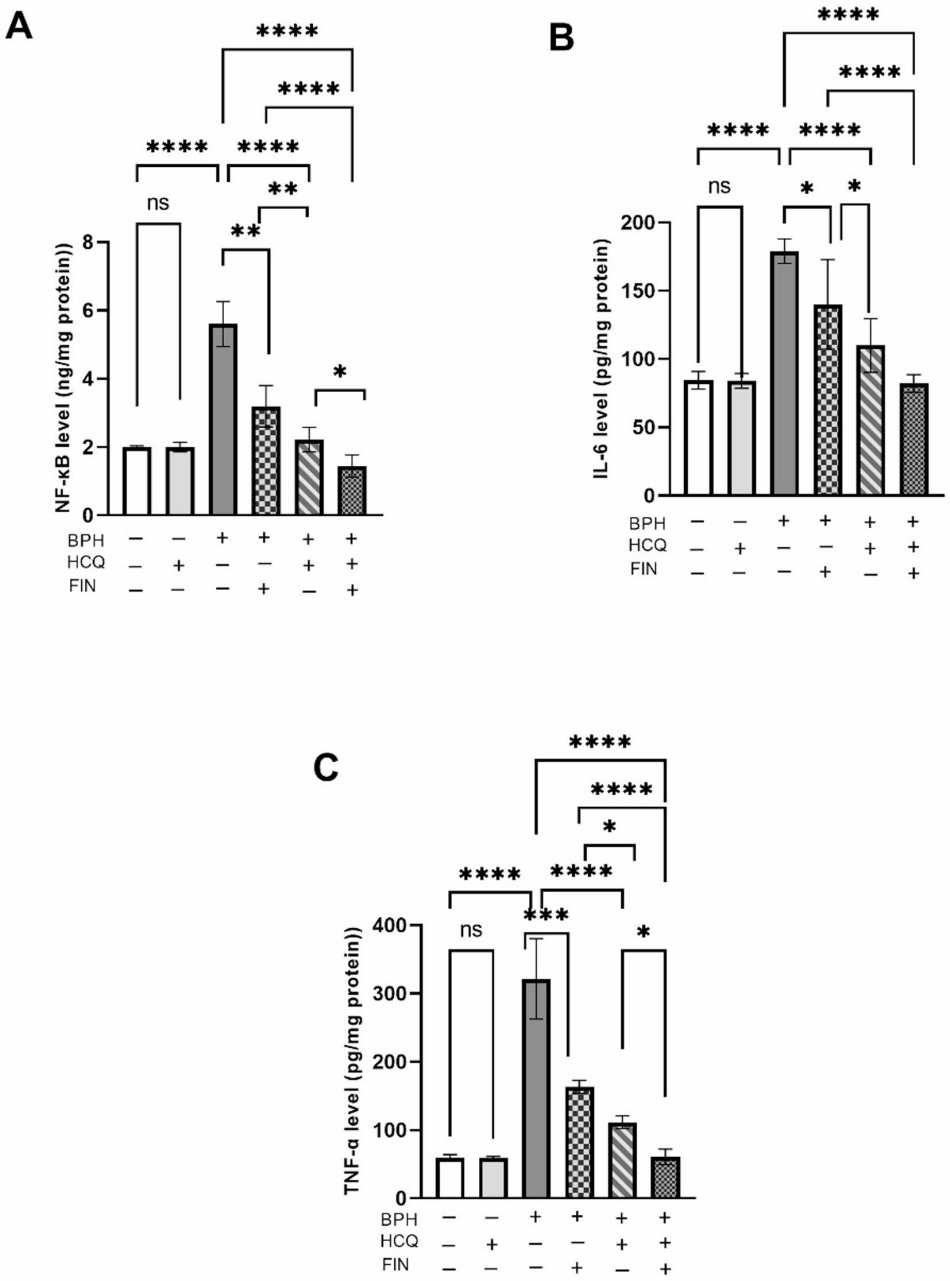
Effect of HCQ, FIN or HCQ/FIN combination on NF-kβ (A), IL-6 (B), TNF-α (C), protein expression levels. One-way ANOVA followed by Tukey’s multiple comparison test was used for statistical analysis. **: Statistically significant at *P* < 0.01, ***: Statistically significant at *P* < 0.001, ****: Statistically significant at *P* < 0.0001.

Testosterone is believed to accelerate the production of pro-inflammatory cytokines, such as TNF-α and IL-6, through the activation of the NF-κB pathway. The levels of key inflammatory markers NF-κB (7 A), IL-6 (7B), and TNF-α (7 C) were significantly elevated in the BPH group compared to normal control. Treatment with HCQ or FIN alone resulted in a reduction in these inflammatory markers, with the combination treatment showing the most pronounced decrease. However, the effect of the combination on IL-6 was not significantly different from the HCQ group (Fig. 4). Furthermore, the FIN/HCQ combination exhibits a synergistic effect, with a CI of 0.36 for IL-6, highlighting the importance of this combined treatment strategy.

### Effect of HCQ, FIN or their combination on ERK1/2, EGFR, and STAT3 signaling pathways

**Fig. 5:**
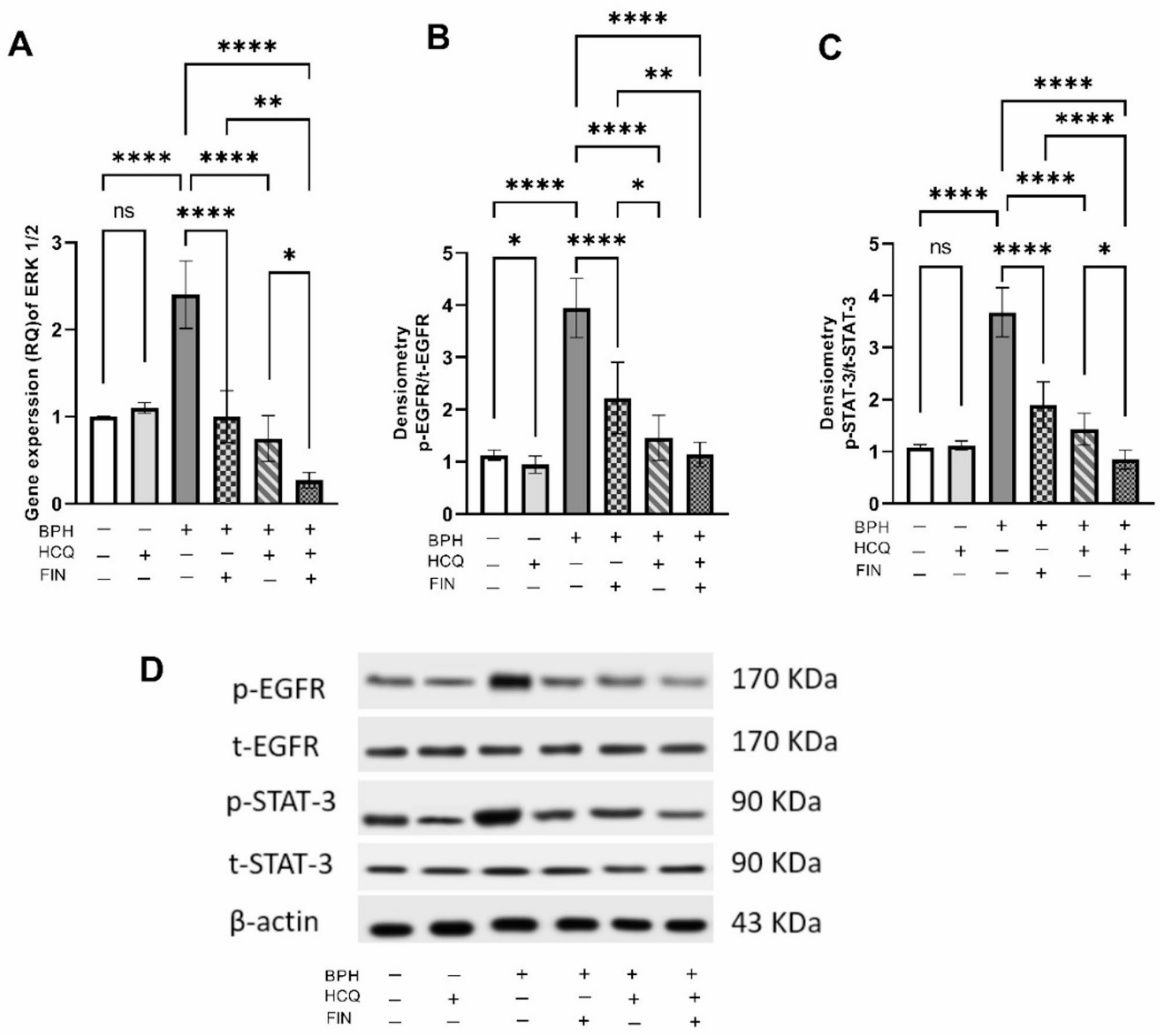
Effect of FIN, HCQ, and a combination of both on the protein expression level of ERK1/2, EGFR, and STAT3 in rats’ prostatic tissue. The expression level of ERK1/2 (A), densitometry analysis of p-EGFR to t-EGFR (B) and p-STAT-3 to t-STAT-3 (C), and western blotting images of EGFR and STAT-3 in both phosphorylated and total forms (D). One-way ANOVA, followed by Tukey’s multiple comparison test, was used for statistical analysis. *: Statistically significant at *P* < 0.05, ***: Statistically significant at *P* < 0.001, ****: Statistically significant at *P < 0.0001.*

The data illustrate the effects of HCQ, FIN, and their combination on key signaling molecules involved in cellular proliferation and survival. In Fig. 5A, ERK1/2 gene expression was significantly upregulated in the BPH group compared to the control, while treatment with HCQ or FIN alone significantly reduced its expression, with the combination treatment showing the most pronounced decrease (*p* < 0.05). In Fig. 5B, the densitometric analysis of p-EGFR/t-EGFR levels showed a significant increase in the BPH group, which was markedly reduced following treatment with HCQ or FIN, with the combination treatment leading to the most significant reduction (*p* < 0.0001). Similarly, in Fig. 5C, p-STAT3/t-STAT3 levels were significantly elevated in the BPH group, whereas both HCQ and FIN treatments effectively reduced phosphorylation levels, with the combination showing the strongest inhibitory effect (*p* < 0.0001). Moreover, the Fig. 5D presents representative Western blot images confirming these findings, demonstrating a substantial reduction in p-EGFR and p-STAT3 levels following treatment. Additionally, the FIN/HCQ combination demonstrates a synergistic effect, with a CI of 0.86 for ERK1/2 and 0.018 for STAT3, emphasizing the significance of this combined therapeutic approach. These results suggest that HCQ or FIN, and particularly in combination, effectively inhibit ERK1/2, EGFR, and STAT3 signaling pathways, which may contribute to their therapeutic potential in BPH management.

### Effect of HCQ,  FIN, or their combination on BPH-induced histopathological alterations 

**Fig. 6:**
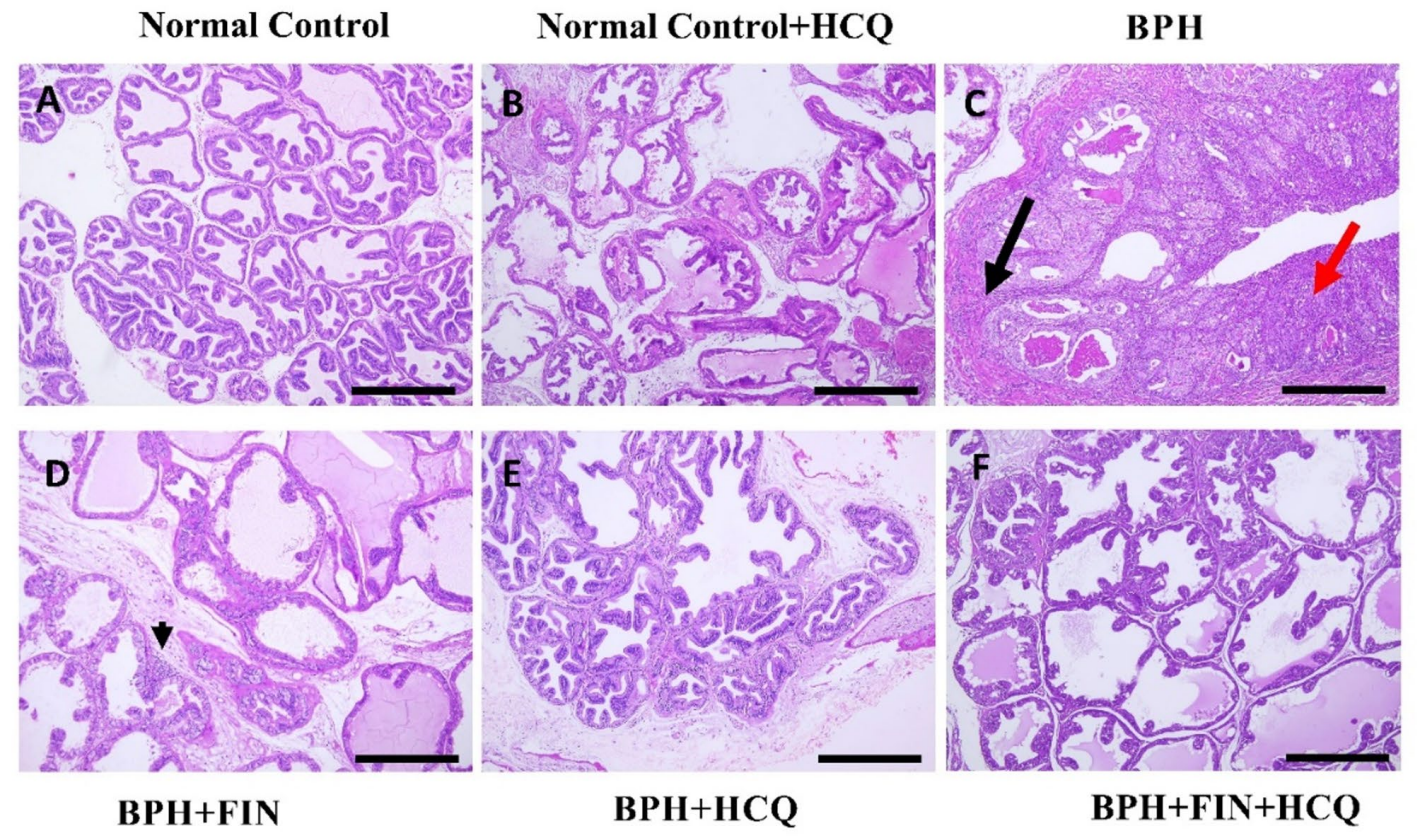
Prostate sections of normal and HCQ (A and B) control groups showed normal, unremarkable architecture, the BPH group showing florid biphasic proliferation with hyperplasia of the glandular (red arrow) and stromal components with intervening inflammation within the stroma (black arrow). The lumen showed inspissated eosinophilic secretions (C). FIN-treated group (D) showing improved architecture and minimal residual inflammation (arrow head), whereas HCQ- (E) and FIN/HCQ- (F) treated groups showing near-normal restoration of the prostate architecture regarding proliferation and size with no significant inflammatory cells (H&E stain, Magnification power = x200, Scale bar = 100 μm).

Grossly, the prostate from the BPH group appeared enlarged and nodular, while those retrieved from the treatment groups were significantly smaller in size. In Fig. 6, microscopy revealed florid biphasic proliferation of the BPH group with hyperplasia (non-neoplastic growth) of glandular (c-red arrow) and stromal components with intervening inflammation within the stroma (c-black arrow). The epithelial hyperplasia is represented here by variably sized glandular structures lined by basal and secretory cells, ranging from cuboidal to small columnar and stratified, as well as papillary structures formed in the lumen of the acini (yellow arrow; Fig. S1). The epithelium showed pale pink cytoplasm with frequent vacuolar degeneration, regular, hyperchromic, centrally located nuclei, and inconspicuous nucleoli. Some glands were dilated with cyst-like formation, often with flat to cuboidal lining. The lumen of the glands showed inspissated eosinophilic secretions and focal inflammatory cells (star; Fig. S1). The stromal element comprises bland spindle cells with round to ovoid nuclei with open chromatin, with occasional infiltrating inflammatory cells, mainly lymphocytes with few segmented leucocytes (black arrow; Fig. S1). Proliferation of both stromal and epithelial cells leads to new glandular budding and branching, with the formation of nodules. On the contrary, the control groups showed no proliferative changes or inflammation. The intact glands were mostly lined by flattened and low cuboidal cells. The treatment groups were closely similar, with no pronounced stromal proliferative changes. However, the glandular element has minimal residual hyperplasia. Some glands appeared cystically dilated with occasional secretory exudate, and interstitial edema. Foci of residual interstitial inflammatory cell infiltrate, especially in the periurethral zone, were noticed more in the FIN group (d-black arrowhead), as well as few luminal leucocytes (star) as shown in Fig. S1. While those with HCQ and combined FIN/HCQ group showed non-significant inflammation.

### Molecular docking

**Fig. 7:**
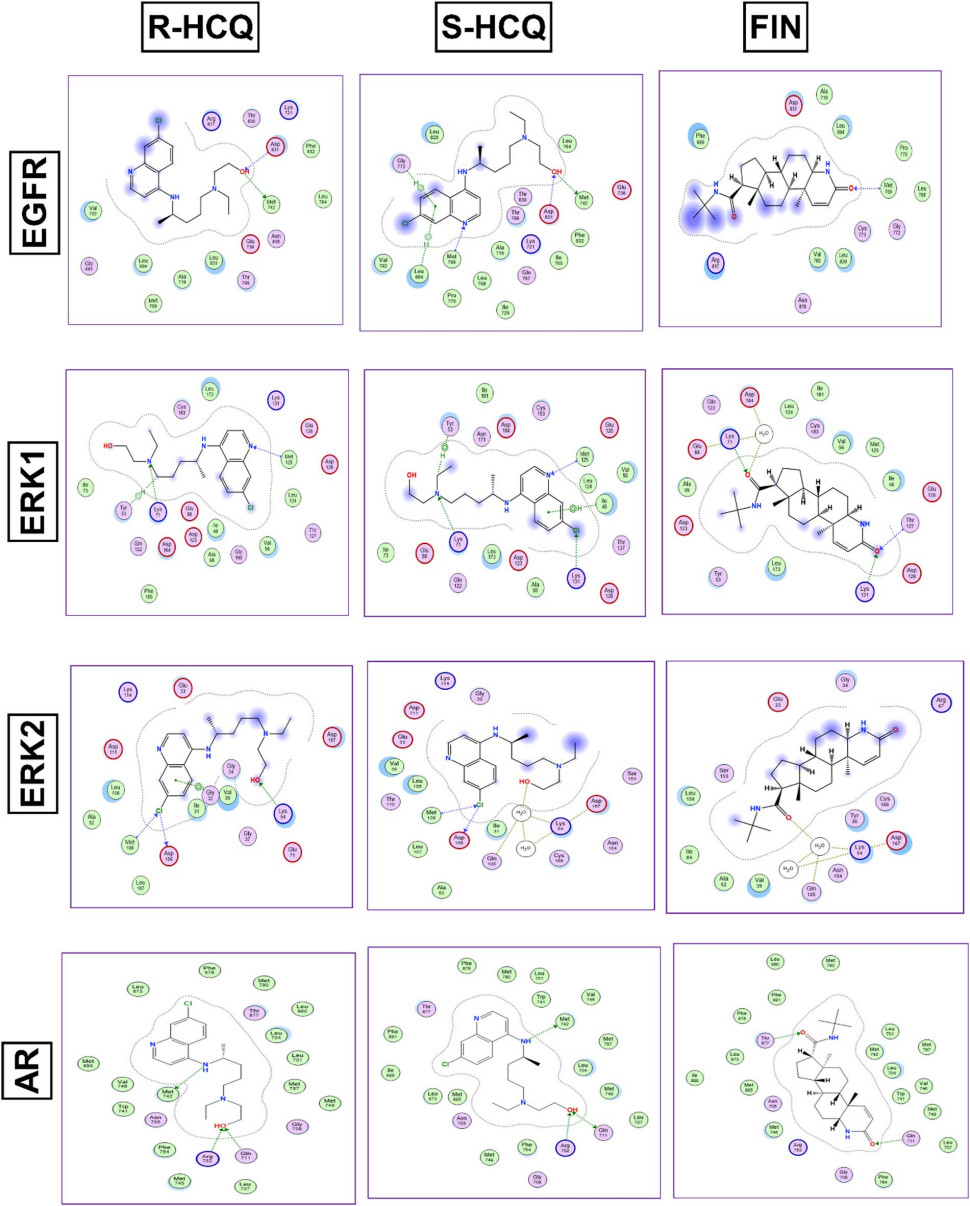
2D diagrams showing the interactions of R-HCQ, S-HCQ and FIN in the EGFR, ERK1/2 and AR binding sites.

**Fig. 8:**
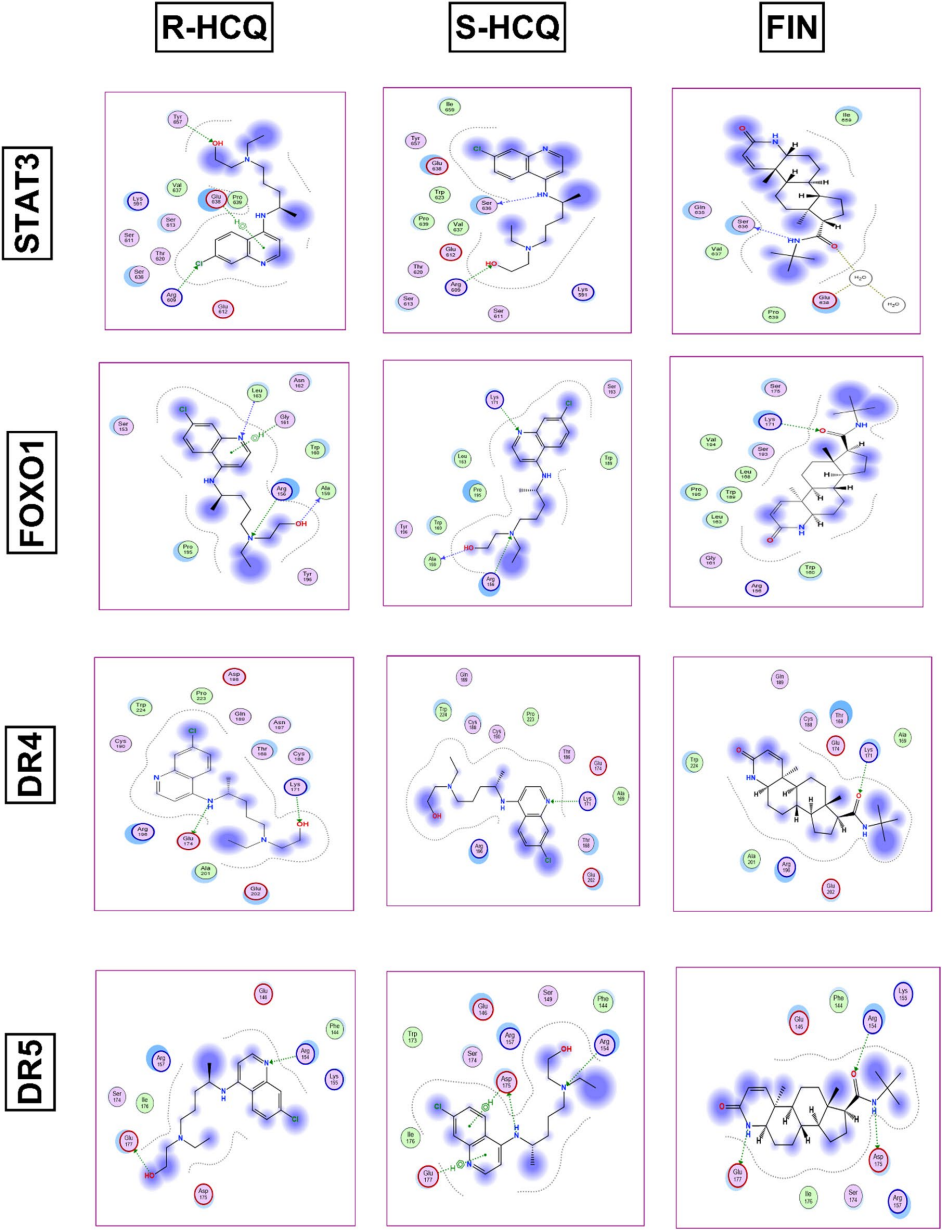
2D diagrams showing the interactions of R-HCQ, S-HCQ, and FIN in the STAT3, FOXO3, and DR4/5 binding sites.

During docking protocol validation, the redocking of the co-crystallized ligands into the active site replicated the same binding interactions observed with the original co-crystallized ligand. This result validates the efficacy of the employed docking protocol in predicting potential binding poses for the compounds. Furthermore, this validation was supported by the small RMSD values between the poses of the native ligand and the re-docked ligand (Table S1). Figures 7 and 8 illustrate the binding pattern of HCQ (both enantiomers) and FIN in the binding site of the studied pathways, represented as 2D diagrams. For more detailed information about the docking scores and the interactions between the docked compounds and the amino acid residues within the binding site of the proteins under study, see the supporting information file (Table S2).

### Protein-protein interactions

**Fig. 9:**
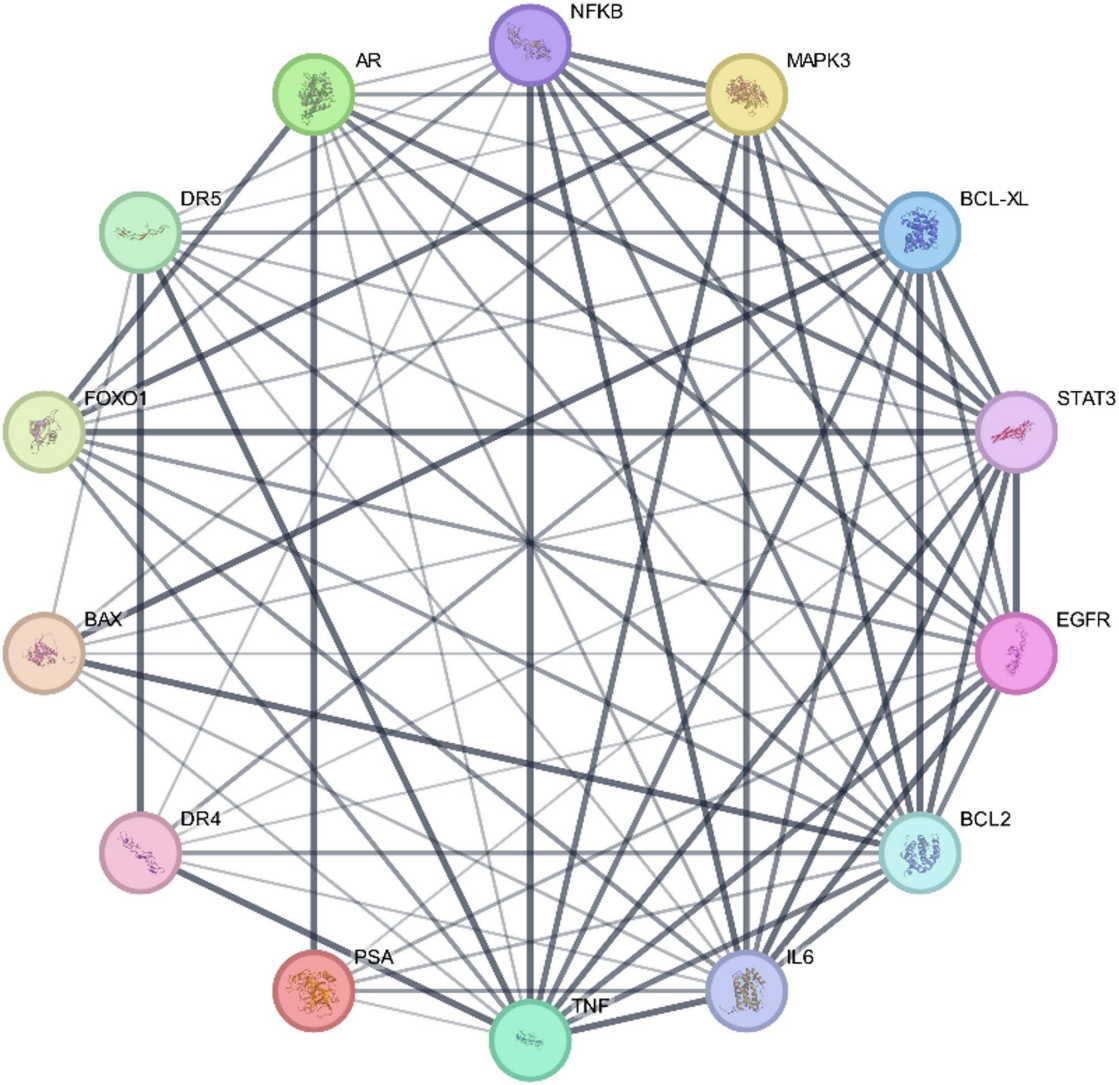
STRING Protein-protein interaction analyses. The network has 72 edges (vs. 28 expected edges); enrichment p-value < 0.0001; clustering coefficient: 0.892; enrichment p-value < 0.001. The thickness of the line denotes the strength of data support.

Utilizing the STRING protein-protein interaction database^46^, we examined the interconnectivity within the chosen targets of HCQ. Employing an interaction score threshold of 0.7 (indicating high confidence), the STRING PPI analysis (Fig. 9) revealed a densely clustered network (clustering coefficient: 0.893) comprising 14 nodes connected by 75 edges (with an anticipated number of edges being 28). This outcome suggests a significantly heightened level of interaction compared to what would be anticipated for a random set of similar size sourced from the genome (enrichment p-value < 0.0001).

## Discussion

Our study introduces a new repurposing approach for HCQ, showcasing its protective effects whether alone or combined with FIN against the progression of testosterone-induced benign prostatic hyperplasia (BPH) in a rat model. Unlike its parent compound, chloroquine, HCQ shows a 40% reduction in toxicity. Importantly, HCQ displays no adverse effects on sperm motility or male sex hormone levels over time. Additionally, HCQ has no long-term impact on male reproductive health, making it a safer therapeutic option for BPH treatment^49,50^. HCQ modulates several signaling pathways, including EGFR^27^, STAT3^28^, and FOXO signaling^29^, as well as exhibiting antiproliferative and apoptotic-inducing activities^30,31^ in cancer therapy and various illnesses. Such multifaceted mechanisms make HCQ a promising candidate in the treatment of BPH.

Based on the above-mentioned assumptions, we investigated the therapeutic potential of HCQ or its combination with FIN in a testosterone-induced BPH rat model. This model is reported to closely mirror the pathological features of BPH observed in humans, providing a robust platform for evaluating the therapeutic potential of our selected drugs^51^. In our study, the SC injection of 3 mg/kg TE for 28 successive days caused a prominent increase in PW and PI, key indicators for BPH progression^52^. This was associated with elevated serum levels of PSA, testosterone, and DHT, along with upregulation of AR gene expression in prostate tissues. DHT activates the AR signaling pathways inducing the expression of target genes involved in prostate cell proliferation and survival^7,8^ and also increasing PSA levels^10^. Our histopathological examinations further confirmed these findings, revealing biphasic proliferation in the stromal and epithelial cells of the prostate.

Conversely, HCQ administration significantly reduced PW and PI, PSA levels, downregulated AR expression, and restored the prostate architecture to near normal. These results were comparable to those obtained with Fin, indicating the potential of HCQ in mitigating the progression of BPH. These results could be attributed to the potential of HCQ in inducing androgen deprivation in prostate tissues, thereby regulating prostate development and growth^32^. Combined therapy showed higher efficacy in reducing these effects than either treatment alone, suggesting a synergistic effect in regulating BPH progression.

Chronic inflammation is a key player in the pathogenesis of BPH, affecting prostate growth and correlating with symptom severity and disease progression^53^. Although the exact mechanisms remain unclarified, the enhanced inflammatory reactions in BPH are closely linked to AR-mediated activation of NF-κB^15^. NF-κB activation triggers the production of pro-inflammatory cytokines, including TNF-α and IL-6, and immune cell infiltration within epithelial and stromal tissues. Elevated IL-6 levels further activate STAT3, a key transcription factor in inflammation and cell proliferation in BPH^11^, creating a feedback loop that exacerbates chronic inflammation^11,54^. Our results align with these data, showing enhanced levels of NF-κB, IL-6, and TNF-α along with upregulated STAT3 protein expression in prostatic tissues. Our histopathological examinations revealed inflammatory cell infiltration in both epithelial and stromal tissues. Notably, HCQ significantly attenuated these inflammatory responses, reducing NF-κB, IL-6, and TNF-α levels and downregulating STAT3 expression. While HCQ’s anti-inflammatory effects have been documented in other diseases^22^, our study is the first to demonstrate its anti-inflammatory potential in BPH, which could partially elucidate its effectiveness in managing this condition. Of note, combined therapy showed enhanced reduction in inflammatory reactions compared to monotherapy.

Moreover, Upregulation of EGFR has been reported in prostatic tissues of BPH patients^55^ and experimental animals^12^. Transactivation of EGFR in prostatic tissues triggers the downstream signaling of several signaling pathways, including the ERK1/2 and STAT3 signaling, which stimulate proliferation and inhibit apoptosis^10,12^. Our findings further support the interplay between the EGFR/ERK/STAT3 signaling axis in BPH development. This is demonstrated by upregulating EGFR protein expression and ERK1/2 gene expression, an essential downstream effector that promotes cell proliferation and survival in BPH-induced rats. Activated ERK1/2 further induces the phosphorylation of STAT3, as previously reported^56^, thereby maintaining chronic inflammation and contributing further to BPH progression. On the contrary, HCQ deactivated the EGFR/ERK/STAT3 pathway, attenuating prostate cell proliferation and inflammation and mitigating BPH development. Combination therapy showed enhanced efficacy in deactivating this signaling pathway.

Furthermore, the disrupted balance between apoptosis and proliferation contributes mainly to the development of BPH^57^. This imbalance is mediated by AR, where its overexpression promotes prostate cell proliferation while suppressing apoptosis through downregulating FOXO1, a transcription factor essential for mitochondrial-mediated and TRAIL-dependent apoptosis transcription^15^. In our BPH model, the overexpressed AR induced a marked suppression in FOXO1 gene expression and STAT3 protein expression, resulting in mitigating mitochondrial-mediated cell death. This was manifested by the decline in the levels of the pro-apoptotic protein “Bax” and an elevation in the anti-apoptotic proteins Bcl-2 and Bcl-XL^58,59^.

Additionally, FOXO1 deactivation disrupts TRAIL signaling, further impairing apoptosis in prostate cells^60,61^. Inconsistency, our findings demonstrated that the suppression in FOXO1 gene expression was coupled with suppressed gene expression of TRAIL death receptors “DR4 and DR5”. Such a shift in the balance of apoptotic regulators likely induces cell survival and proliferation of prostate cells. Noteworthy, HCQ restored this balance by modulating the crosstalk between AR and FOXO1, enhancing TRAIL-mediated and intrinsic apoptosis pathways, and inhibiting proliferation. Such data add further supportive evidence that HCQ, particularly when combined with Fin, can modulate the progression of BPH.

Finding the above mentioned significant effect of HCQ and FIN on BPH-induced rats, it was noteworthy to gain insight into the ability of these drugs to modulate, not only the expression, but also the activity of STAT3/FOXO1/TRAIL and EGFR/ERK/AR pathways’ members through a molecular docking study. The obtained docking patterns and scores demonstrated both HCQ and FIN’s ability to interact with the reported key amino acids^40,41,62–67^. By analyzing the produced docking poses, it was observed that HCQ exhibited more hydrogen and/or halogen bond interactions than FIN across all the pathway members, except for STAT3, where both compounds showed comparable interactions. Additionally, for ERK1 and DR5, FIN interacted via more hydrogen bonds than HCQ within the binding pocket. Given that HCQ is available as a racemic mixture, it was essential to dock both enantiomers. This analysis revealed that both enantiomers bind similarly to most of the proteins under study, confirming the activity of the racemic mixture.

**Fig. 10:**
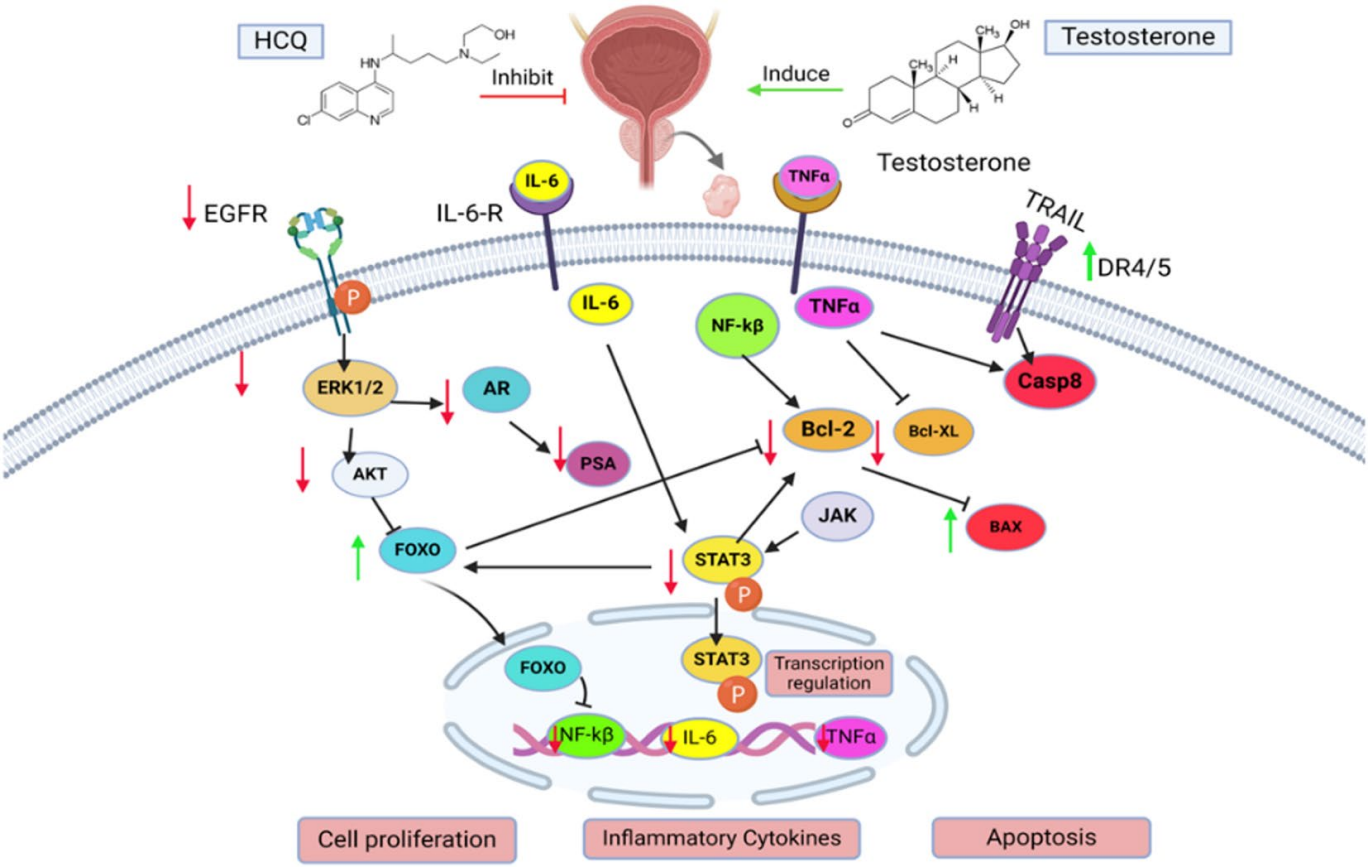
HCQ alone or in combination with FIN alleviated BPH through targeting STAT3/FOXO1/TRAIL and EGFR/ERK/AR signaling pathways.

In conclusion, our study demonstrates, for the first time, the effectiveness of HCQ in mitigating the progression of testosterone-induced BPH. HCQ targeted multiple signaling pathways in BPH-induced rats, including AR-mediated signaling pathways linked to chronic inflammation, and dysregulated cell proliferation and apoptosis. HCQ modulated the EGFR/ERK/STAT3 signaling axis and restored the balance between apoptosis and proliferation by enhancing FOXO1-mediated pathways. Combination therapy exhibited higher efficacy than monotherapy, suggesting a synergistic effect in regulating BPH progression (Fig. 10). Moreover, the molecular docking study underscores the potential of HCQ and FIN as activity modulators of the STAT3/FOXO1/TRAIL and EGFR/ERK/AR signaling pathways’ members, with HCQ showing more interactions than FIN across all the pathways members, except for STAT3, where both compounds showed comparable interactions. Overall, this study contributes to a more profound comprehension of the therapeutic potential of HCQ in addressing BPH.

Consequently, HCQ is a promising therapeutic agent for BPH, providing a new approach to managing this condition whether as a monotherapy or combined with Fin. However, further clinical studies are required to verify our data, thus opening new avenues for its translational potential in BPH treatment.

## Electronic supplementary material

Below is the link to the electronic supplementary material.

Supplementary Material 1

Supplementary Material 2

## Data Availability

Data are available upon reasonable request from the corresponding author.

## Abbreviations

AR: Androgen receptor
BPH: Benign prostatic hyperplasia
DHT: Dihydrotestosterone
DR: Death receptor
EGFR: Epidermal growth factor receptor
ELISA: Enzyme-linked immunosorbent assay
FIN: Finasteride
FOXO: Forkhead Box O
HCQ: Hydroxychloroquine
HRP: Horseradish peroxidase
IL: Interleukin
LUTS: Lower urinary tract symptoms
NF: Nuclear factor
PSA: Prostate-Specific Antigen
SD: Standard deviation
STAT: Signal Transducer and Activator of Transcription
TE: Testosterone enanthate
TNF: Tumor necrosis factor
