# Supplementary material for: Hydroxychloroquine modulates the progression of experimentally induced benign prostatic hyperplasia in rats via targeting EGFR/ERK/STAT3 and AR/FOXO1/TRAIL pathways: computational and in vivo studies
Source: Sci Rep. 2025 Jun 20;15:20118. doi: 10.1038/s41598-025-04267-y (PMC12181434; doi:10.1038/s41598-025-04267-y)
Supplement: Supplementary file 1 — Supplementary Material 1 [file 41598_2025_4267_MOESM1_ESM.pdf]

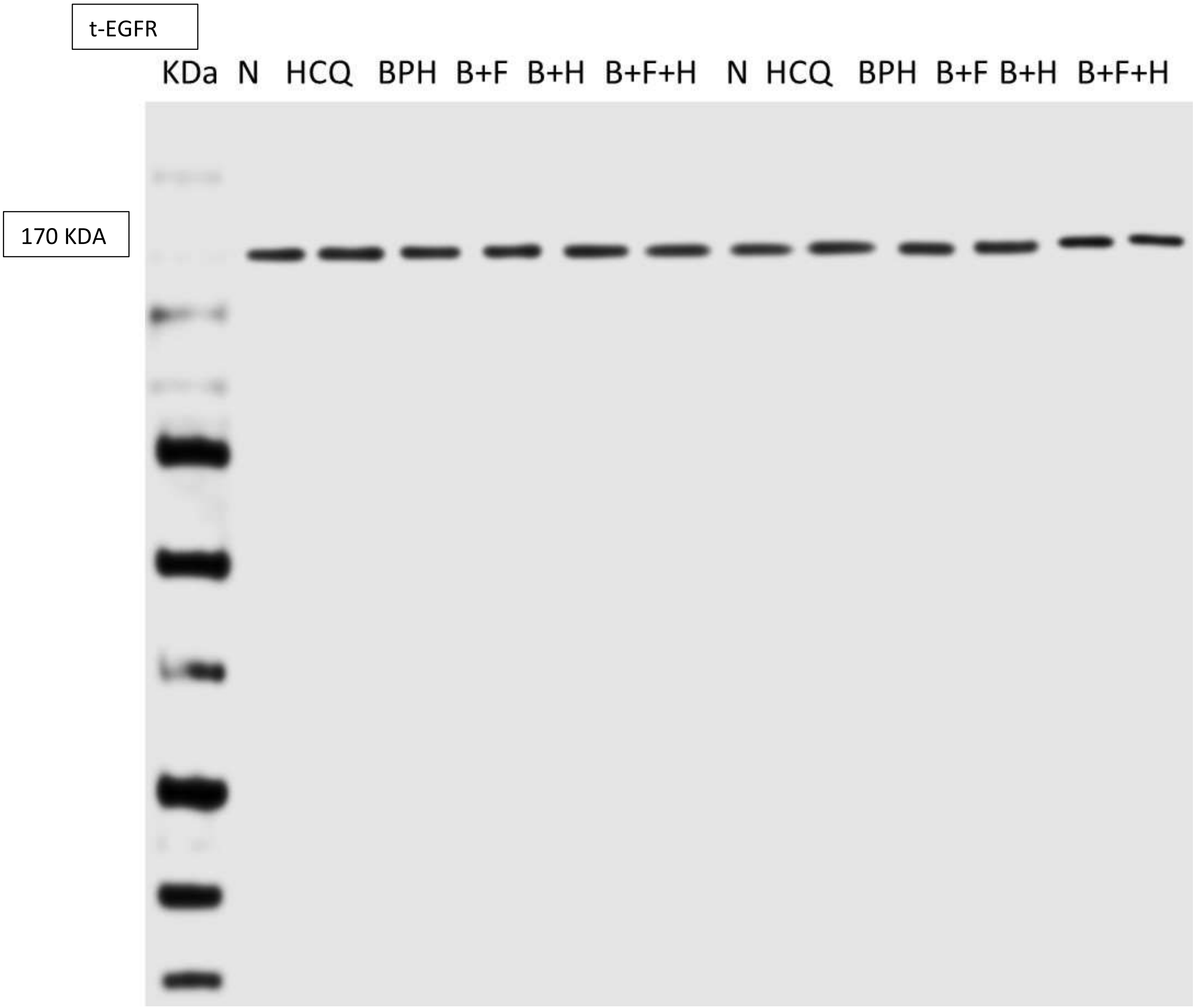
t-EGFR
KDa N HCQ BPH B+F B+H B+F+H N HCQ BPH B+F B+H B+F+H
170 KDA

P-STAT-3

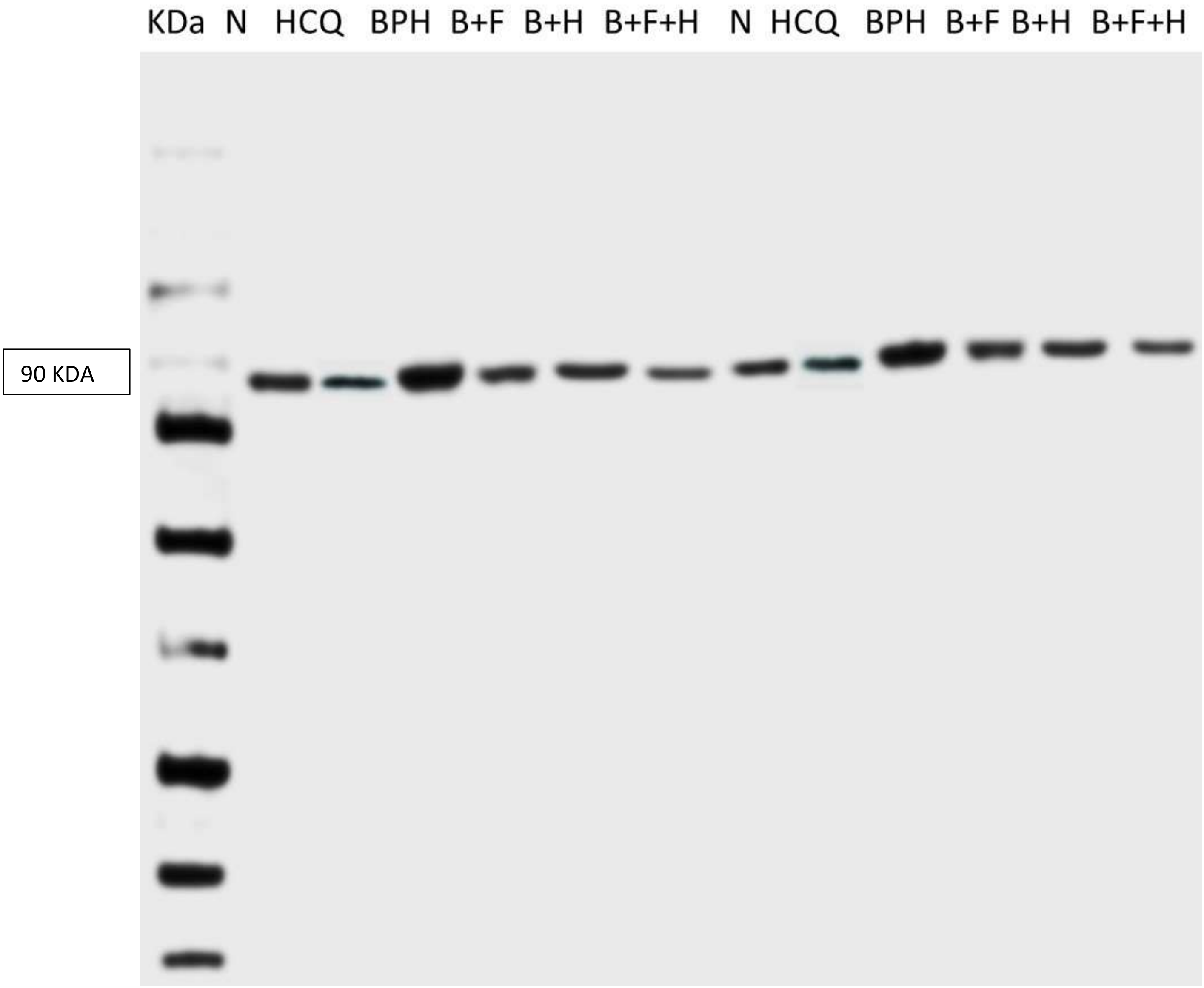

P-EGFR

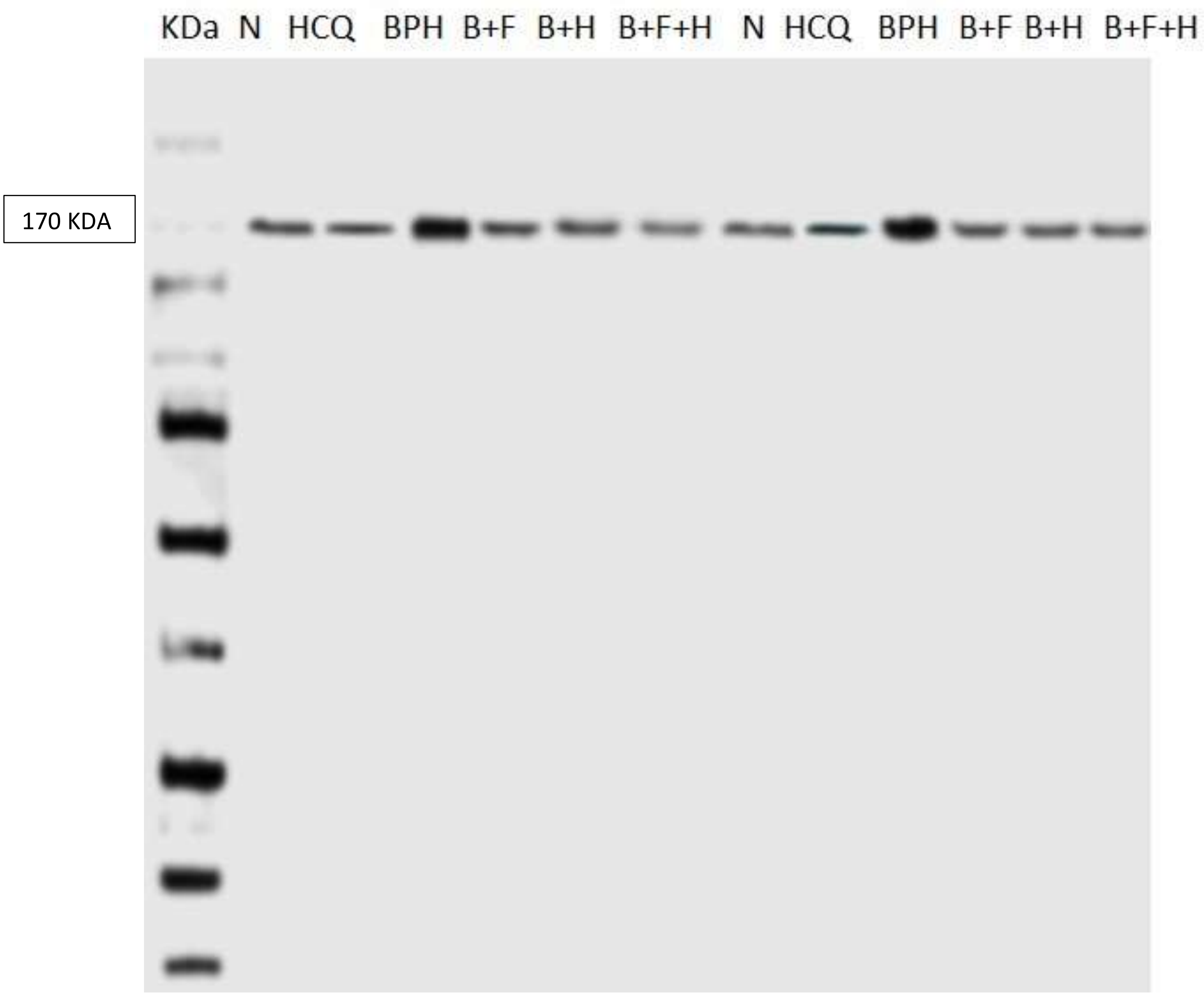

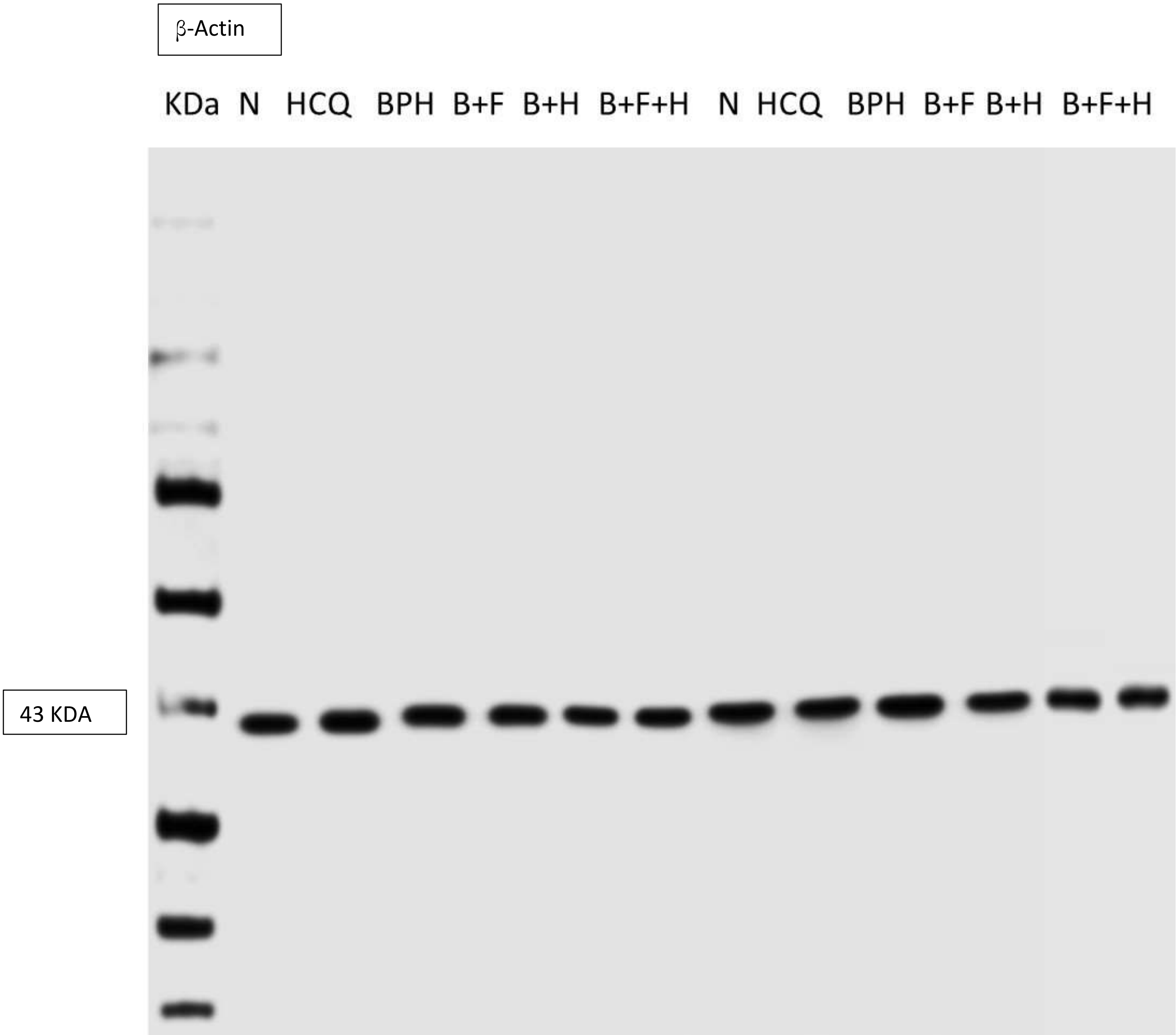
β-Actin
KDa N HCQ BPH B+F B+H B+F+H N HCQ BPH B+F B+H B+F+H
43 KDA

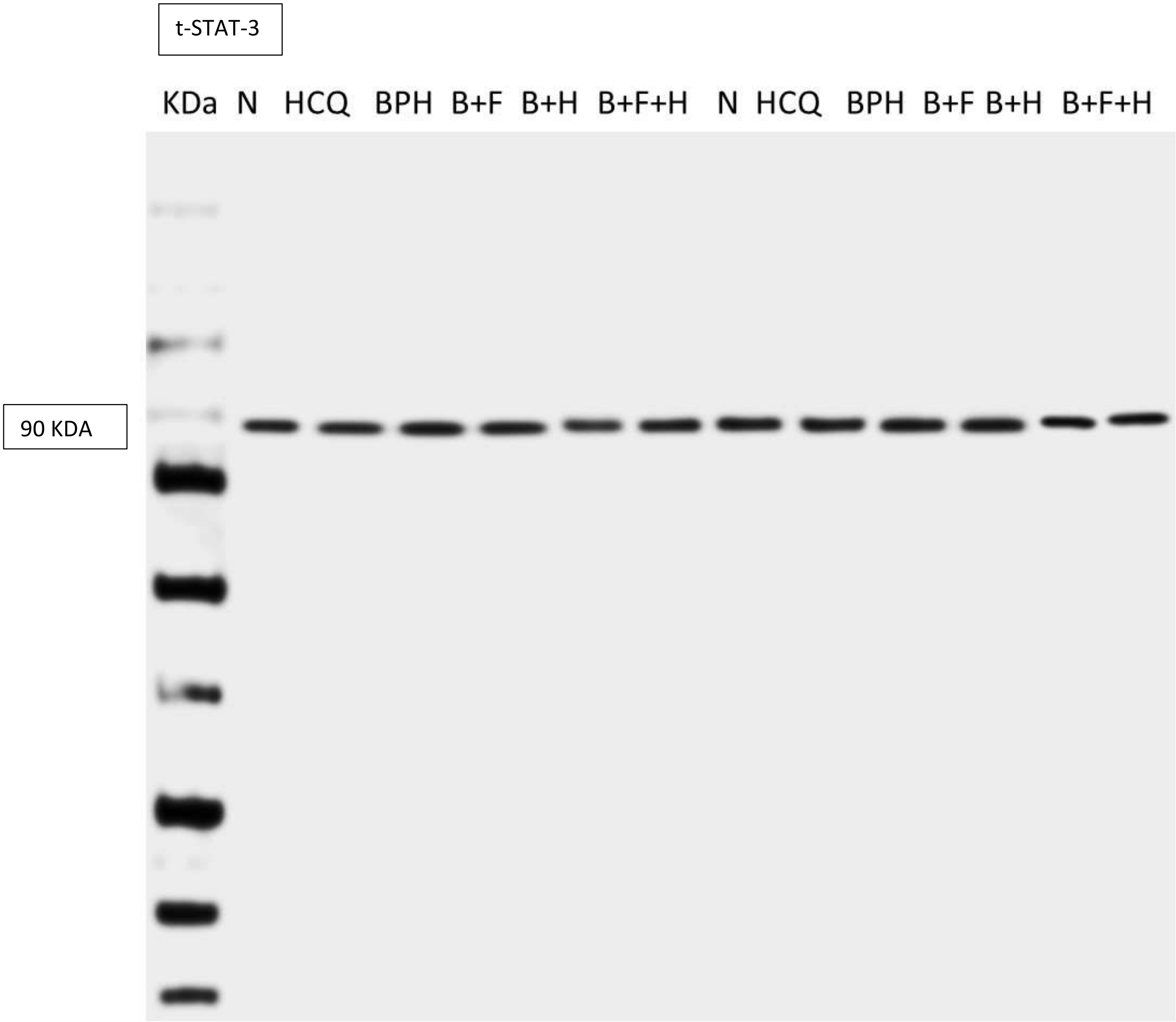
t-STAT-3
KDa N HCQ BPH B+F B+H B+F+H N HCQ BPH B+F B+H B+F+H
90 KDA
