# Supplementary material for: Hydroxychloroquine modulates the progression of experimentally induced benign prostatic hyperplasia in rats via targeting EGFR/ERK/STAT3 and AR/FOXO1/TRAIL pathways: computational and in vivo studies
Source: Sci Rep. 2025 Jun 20;15:20118. doi: 10.1038/s41598-025-04267-y (PMC12181434; doi:10.1038/s41598-025-04267-y)
Supplement: Supplementary file 2 — Supplementary Material 2 [file 41598_2025_4267_MOESM2_ESM.pdf]

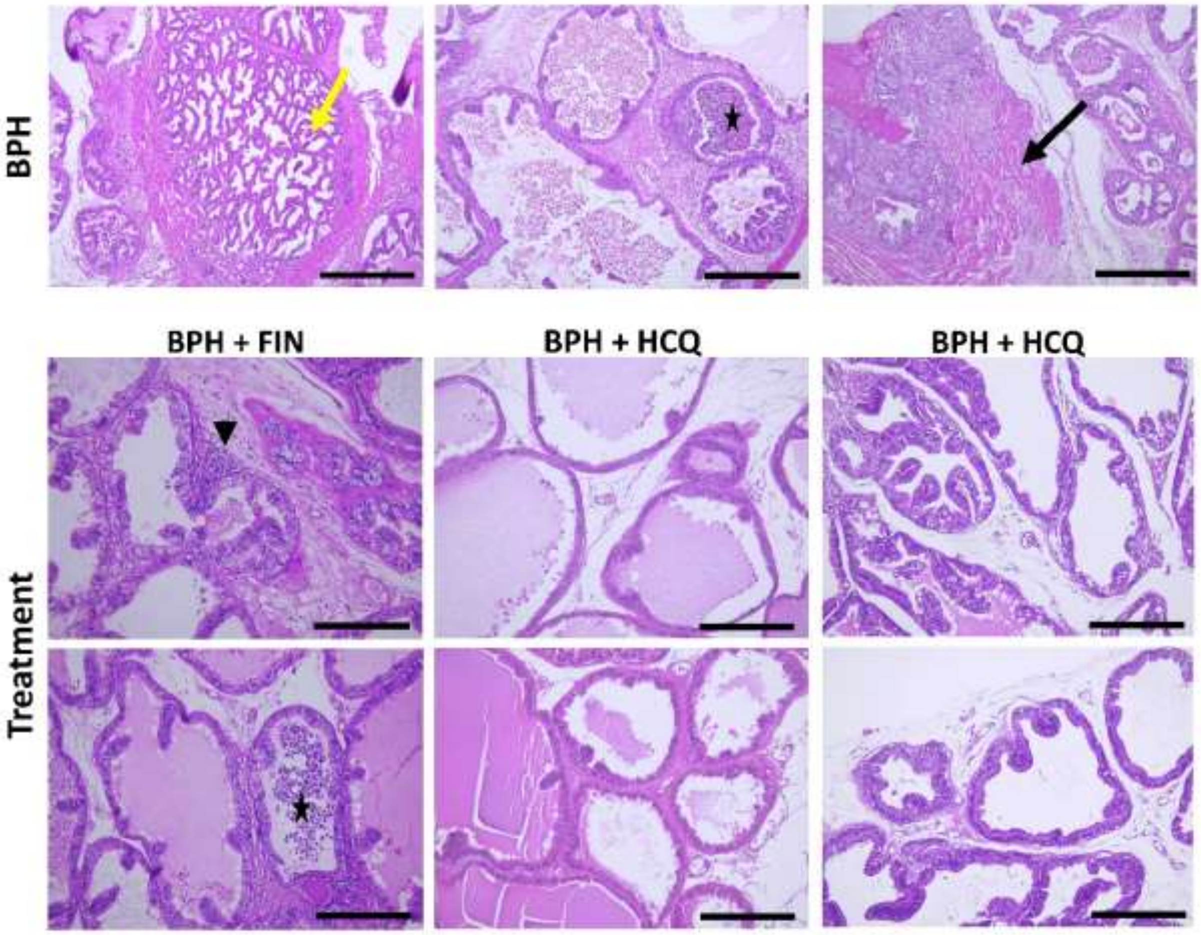

Figure S1. Prostate sections of (Upper Panel) BPH group showing papillary structures in the lumen of the acini (yellow arrow). Some glands were dilated with cysts-like formation, with low cuboidal lining. The glands lumen showed inspissated eosinophilic secretions and focal inflammatory cells (star supplementary figure). The stromal element is composed of bland spindle cells with round to ovoid nuclei with open chromatin with occasional infiltrating inflammatory cells mainly lymphocytes with few segmented leucocytes (black arrow) (H&E stain, Magnification power = x200, Scale bar = 100μm). Lower Panel: The FIN treated group showing residual interstitial inflammation (black arrowhead), as well as luminal leucocytes (star). The HCQ and FIN/HCQ combined treated group showing no significant inflammatory cells (H&E stain, Magnification power = x400, Scale bar = 50μm).

Table S1. Results of the validation of the docking protocol setup.

PDB ID	Protein	Co-crystallized ligand	RMSD (Å)	Docking score of the co-crystallized ligand (kcal/mol)
1M17	EGFR	Erlotinib	1.3504	-8.7581
4QTB	ERK1	SCH772984	0.1345	-15.5713
6SLG	ERK2	Tizaterkib	0.2081	-10.6978
1T7R	AR	5-α-dihydrotestosterone	0.0961	-10.1073
6NJS	STAT3	SD-36	1.2075	- 10.4703

Table S2. Molecular docking data of R-hydroxychloroquine, S-hydroxychloroquine and Finasteride in pathway members' active site.

**Compound**	**R-hydroxychloroquine**			**S-hydroxychloroquine**			**Finasteride**		
**Protein**	**Score (S) (kcal/mol)**	**Interacting amino acids**	**Type of interaction**	**Score (S) (kcal/mol)**	**Interacting amino acids**	**Type of interaction**	**Score (S) (kcal/mol)**	**Interacting amino acids**	**Type of interaction**
**EGFR**	-6.5532	Met742<br>Asp831	H-bond<br>H-bond	-6.9715	Met742<br>Met769<br>Asp831<br>Leu694<br>Gly772	H-bond<br>H-bond<br>H-bond<br>H-π stacking<br>H-π stacking	-5.4640	Met769	H-bond
**ERK1**	-7.3045	Lys71<br>Met125<br>Tyr53	H-bond<br>H-bond<br>H-π stacking	-6.8385	Lys71<br>Met125<br>Lys131<br>Ile48<br>Tyr53	H-bond<br>H-bond<br>Halogen-bond<br>H-π stacking<br>H-π stacking	-6.8443	Lys71<br>Thr127<br>Lys131<br>Glu88&Asp184	H-bond<br>H-bond<br>H-bond<br>H-bond (Water-mediated)
**ERK2**	-6.8876	Lys54<br>Asp106<br>Met108<br>Val39	H-bond<br>Halogen-bond<br>Halogen-bond<br>H-π stacking	-6.738	Lys54&Gln105<br>Asp106<br>Met108	H-bond (Water-mediated)<br>Halogen bond<br>Halogen bond	-6.3266	Lys54&Gln105	H-bond (Water-mediated)
**AR**	-7.3190	Gln711<br>Met742<br>Arg752	H-bond<br>H-bond<br>H-bond	-8.4337	Gln711<br>Met742<br>Arg752	H-bond<br>H-bond<br>H-bond	-6.7231	Gln711<br>Thr877	H-bond<br>H-bond
**STAT3**	-5.3816	Arg609<br>Tyr657<br>Glu638	Halogen-bond<br>H-bond<br>H-π stacking	-5.2226	Arg609<br>Ser636	H-bond<br>H-bond	-4.5495	Ser636<br>Glu638	H-bond<br>H-bond (Water-mediated)
**FOXO1**	-5.2017	Arg156<br>Ala159<br>Lys163<br>Gly161	H-bond<br>H-bond<br>H-bond<br>H-π stacking	-5.0546	Arg156<br>Ala159<br>Lys171	H-bond<br>H-bond<br>H-bond	-5.0752	Lys171	H-bond

**Compound**	**R-hydroxychloroquine**			**S-hydroxychloroquine**			**Finasteride**		
**Protein**	**Score (S) (kcal/mol)**	**Interacting amino acids**	**Type of interaction**	**Score (S) (kcal/mol)**	**Interacting amino acids**	**Type of interaction**	**Score (S) (kcal/mol)**	**Interacting amino acids**	**Type of interaction**
**DR4**	-5.7000	Lys171<br>Glu174	H-bond<br>H-bond	-5.8205	Lys171	H-bond	-5.398	Lys171	H-bond
**DR5**	-5.0872	Arg154<br>Glu177	H-bond<br>H-bond	-5.1635	Arg154<br>Asp175<br>Asp175<br>Glu177	H-bond<br>H-bond<br>H-π stacking<br>H-π stacking	-5.0674	Arg154<br>Asp175<br>Glu177	H-bond<br>H-bond<br>H-bond
